# Degradation of the neonicotinoid pesticide imidacloprid by electrocoagulation and ultrasound

**DOI:** 10.1038/s41598-024-59568-5

**Published:** 2024-04-17

**Authors:** Ivan Halkijevic, Katarina Licht, Vanja Kosar, Lucija Bogdan

**Affiliations:** 1 Faculty of Civil Engineering, University of Zagreb, Kaciceva 6, Zagreb, Croatia; 2 Faculty of Chemical Engineering and Technology, University of Zagreb, Savska 16, Zagreb, Croatia

**Keywords:** Environmental chemistry, Pollution remediation, Chemical engineering

## Abstract

Imidacloprid is still a widely used neonicotinoid insecticide that is banned in many countries because of the associated environmental risks. Due to the inefficiency of conventional wastewater treatments for pesticide removal, new treatment methods are being investigated. Electrochemical methods, including electrocoagulation (EC), seem to be promising alternatives considering their effectiveness in removing various pollutants from wastewater. The aim of this study was to investigate the effects of electrode material, current density, ultrasound, and operation time on the efficiency of imidacloprid removal from a model solution by EC. The combination of aluminum electrodes and 20 A of applied current for 20 min resulted in total imidacloprid degradation. A simplified energy balance was introduced as a form of process evaluation. Combining ultrasound with EC resulted in 7% to 12% greater efficacy than using only EC.

## Introduction

Neonicotinoids are a group of insecticides used on a variety of crops as well as in gardens, lawns, and households ^1^. These compounds are selective, have low toxicity to vertebrates, and are highly effective against arthropods, sucking insects, and chewing insects ^2^. Imidacloprid was the first neonicotinoid insecticide to be approved for commercial use in the 1990s. It is used in agriculture and horticulture to control grubs and termites and to treat fleas and ticks in animals ^3^. Although neonicotinoids have excellent insecticidal properties, they are also characterized by high polarity, solubility, and photostability, which can cause them to persist as pollutants in the environment ^4^. Some of these insecticides, including imidacloprid, clothianidin, and thiamethoxam, have been linked to declines in bee populations and pose a risk to the entire ecosystem ^2,5,6^. Due to the associated environmental risks, the use of imidacloprid has recently been banned by European Union regulations (EU, 2018/783). However, the presence of neonicotinoids in aquatic environments due to agricultural runoff and effluents ^7,8^ indicates the need for an efficient remediation method. Due to their chemical properties, neonicotinoids are more resistant to degradation, and conventional water treatment based on biological processes is not sufficient to fully mineralize them ^9^. Several methods, especially advanced oxidation processes, such as photolysis, photocatalysis, photo-Fenton, ozonation, electrocatalytic oxidation, and electro-Fenton oxidation, have been successfully used for the removal of imidacloprid from water ^10–12^.

In recent years, electrochemical processes have gained attention as efficient methods for treating waters containing various pollutants, such as heavy metals, selenium, mineral oils, dyes, microorganisms, and pesticides ^10,13–17^. Electrocoagulation (EC) is a widely used electrochemical method for water and wastewater treatment ^17^. This method combines the advantages of coagulation, flotation, and electrochemistry ^18–20^. Electrocoagulation uses sacrificial electrodes to generate flocculants through electrooxidation ^15^. This process works by dissolving the metal anode due to the applied current density, leading to the in situ formation of the coagulant, as shown in Eq. 1^14,21^. These coagulant species can then react with the organic compounds in wastewater to form flocs (Eq. 4) that can be easily removed by sedimentation or flotation. At the same time, oxygen evolves at the anode (Eq. 2), and hydrogen evolves at the cathode (Eq. 3), allowing the removal of contaminants by flotation ^18^.

1 $$\begin{array}{c}M\to {{\text{M}}}^{{\text{n}}+}+n{{\text{e}}}^{-}\end{array}$$

2 $$\begin{array}{c}2{{\text{H}}}_{2}O\to 4{{\text{H}}}^{+}+{{\text{O}}}_{2}+4{e}^{-}\end{array}$$

3 $$\begin{array}{c}2{{\text{H}}}_{2}O+2{e}^{-}\to {{\text{H}}}_{2}+2{{\text{OH}}}^{-}\end{array}$$

4 $$\begin{array}{c}{{\text{M}}}^{{\text{n}}+}+n{{\text{OH}}}^{-}\to {{\text{M}}({\text{OH}})}_{{\text{n}}}\end{array}$$

Aluminum and iron electrodes are most commonly used due to their efficiency, availability, low toxicity, and relatively low price ^19^. The efficiency of electrocoagulation for removing organic compounds depends on several factors, such as the initial concentration of the organic compound, the current density, the pH, the electrolysis time, and the electrode material. The EC process has several advantages, including the use of minimal chemical reagents, simple equipment, low sludge production, and low concentrations of secondary pollutants ^15,18,20^. The main disadvantages of EC include the passivation of electrodes due to the formation of oxide layers on the surface of the electrodes, the need for periodic replacement of sacrificial anodes due to material loss, the potential need for posttreatment due to the high metal ion concentrations generated during the process, and the high electricity consumption costs in areas with limited access to electricity ^18,22^. There are several ways to overcome these disadvantages, including combining EC with other methods, such as ultrasound (US). Combining US with EC can help minimize electrode passivation due to the mechanical effects of US cavitation ^23^. Additionally, during acoustic cavitation, highly reactive oxygen species (e.g., hydrogen (H·) and hydroxyl radicals (·OH)) are generated, as shown in Eq. (5); these species oxidize or reduce organic and inorganic molecules present in water, depending on their reactivity. The reactions and products of acoustic cavitation vary greatly depending on the specific molecules involved and the conditions of the cavitation, such as temperature, pressure, and the presence of other substances in the water. Usually, a mixture of many different reactions occurs simultaneously, and the outcomes can be quite complex ^24^. Different molecules can easily enter cavitation bubbles and become exposed to the extreme conditions of collapsing bubbles ^25,26^.

5 $$\begin{array}{c}{{\text{H}}}_{2}O+)))\to \cdot OH+\cdot H\end{array}$$

The complexity of these reactions especially relates to the simultaneity of electrochemical processes and acoustic cavitation.

There are a few studies on the removal of imidacloprid from water by electrocoagulation, but the efficacy of the combined method of electrocoagulation and ultrasound has not been previously investigated. In the study by Nasser and Nader ^10^, the removal efficiency of imidacloprid and chemical oxygen demand from 100 mL of solution using the electrocoagulation process were 95% and 89.5%, respectively, with Fe electrodes at 60 min and 80.8% and 73.1%, respectively, with Al electrodes at 90 min, at a current density of 18.5 mA/cm^2^, and with the addition of 1 g/L NaCl as the electrolyte. The initial pH ranged from 2.4 to 10, while the highest efficiencies were achieved at pH 6.9. This study showed that increasing the temperature had a negative effect on the removal efficiency, while the maximum efficiency was reached at 20 °C. In a study by Abdel-Gawad et al. ^13^, 98% imidacloprid removal was achieved using Fe electrodes under the following operating conditions: initial pH of 6, current density of 1 mA/cm^2^, 500 mL of treated water, electrolysis time of 10 min, initial pesticide concentration of 0.5% and NaCl concentration of 1 g/L.

The application of EC treatment has been investigated for the removal of various pesticides, including acetamiprid, glyphosate, malathion, oxyfluorfen, diazinon, chlorpyrifos and coragen. A comparison of the efficiencies and operating parameters can be found in Table 1. An article by Biswas and Goel ^21^ provides an overview of these studies as well as a general discussion of removal mechanisms, kinetics, modeling, influencing factors, and sludge characterization of pesticide removal by electrocoagulation and electrooxidation.

**Table 1:** Review of studies on pesticide removal from water using electrocoagulation.

Pesticide	Acetamiprid	Glyphosate	Malathion	Oxyfluorfen	Diazinon	Chlorpyrifos	Coragen
Operating Volume	0.5 L	0.5 L	0.6 L	4 L	1 L	1 L	3 L
Anode Material/Electrode distance (Total Surface Area)	Al/n.a. (478 cm^2^)	Al/n.a. (25 cm^2^)	Al/3 cm (25 cm^2^)	Fe/n.a. (121 cm^2^)	Fe/1.5 cm (100 cm^2^)	Fe/3 cm (99.6 cm^2^)	Fe/2 mm (312 cm^2^)
Current Density (Voltage)	50 A/m^2^	60 A/m^2^	(8 V)	35.5 A/m^2^	500 A/m^2^	36 A/m^2^	75 Am^2^
Operating Time	60 min	60 min	50.94 min	96 h	60 min	50 min	150 min
Electrolyte	0.75 g/L NaCl	1.0 g/L NaCl	12.3 mL of NaCl solution	10 g/L of Na_2_SO_4_	KCl (n.a.)	NaCl (n.a.)	2.5 mg/L of NaCl
Initial Concentration	2.14 mg/L	100 mg/L	20 mg/L	100 mg/L	100 mg/L	800 mg/L	(n.a.)
pH	7.77	6.7	9	7.8—8.2	3	7	7
Removal Efficiency	97.60%	94.25%	96.82%	25%	99.99%	99.99%	79%
Reference	^27^	^28^	^29^	^30^	^31^	^32^	^15^

The published studies are generally characterized by a relatively long operating time, a relatively small reactor volume and the use of electrolytes, which can contribute to the formation of harmful byproducts during the decomposition of the pollutants present ^33–35^.

In this paper, three different electrode materials, current densities, and treatment times were tested for the removal of imidacloprid via EC. A statistical experimental design was used to determine the most influential parameters and their optimal combination. In addition, a series of experiments was carried out using EC treatment combined with US. A kinetic study was also conducted for the combination of parameters that yielded the highest imidacloprid degradation.

## Materials and methods

### Statistical experimental design

The design of experiments (DoE) approach was used in this study. The goal of DoE is to minimize the number of experiments required to obtain the desired information and to maximize the amount of information obtained from each experiment. A face-centered central composite design of the response surface methodology (RSM) was used to develop a statistical model and define the optimal conditions for the electrochemical removal of imidacloprid from water. The individual and interaction effects of the applied electric current, operation time and electrode material on the process efficiency were investigated. The factors were studied at three levels: 5, 12.5, and 20 A of current; 5, 12.5 and 20 min of operation time; and three different materials—aluminum (Al), copper (Cu) and iron (Fe), Table 2. The efficiency of the process was chosen as the response and calculated according to Eq. (6), where *c*_*0*_ and *c*_*t*_ are the initial and final concentrations of imidacloprid, respectively.

**Table 2:** The experimental matrix and corresponding efficiencies.

		Factor A	Factor B	Factor C	Response
Std	Run	Current (A)	Time (min)	Material	Efficiency (%)
24	1	20	20	Cu	87.04
21	2	5	5	Cu	2.16
10	3	12.5	12.5	Fe	94.04
11	4	5	5	Al	0.39
27	5	12.5	5	Cu	7.43
15	6	5	12.5	Al	1.88
16	7	20	12.5	Al	42.67
25	8	5	12.5	Cu	10.08
19	9	12.5	12.5	Al	15.7
5	10	5	12.5	Fe	9.33
23	11	5	20	Cu	13.87
20	12	12.5	12.5	Al	18.22
29	13	12.5	12.5	Cu	48.1
14	14	20	20	Al	100
28	15	12.5	20	Cu	52.35
6	16	20	12.5	Fe	43.05
1	17	5	5	Fe	4.48
4	18	20	20	Fe	96.51
13	19	5	20	Al	1.03
2	20	20	5	Fe	38.33
3	21	5	20	Fe	14.32
22	22	20	5	Cu	9.41
9	23	12.5	12.5	Fe	83.85
7	24	12.5	5	Fe	7.26
12	25	20	5	Al	10.4
30	26	12.5	12.5	Cu	50.14
26	27	20	12.5	Cu	63.42
17	28	12.5	5	Al	3.33
18	29	12.5	20	Al	24.69
8	30	12.5	20	Fe	34.91

6 $$Efficiency \left(\%\right)=\left(1-\frac{{c}_{t}}{{c}_{0}} \right)\times 100$$

### Experimental setup

**Figure 1:**
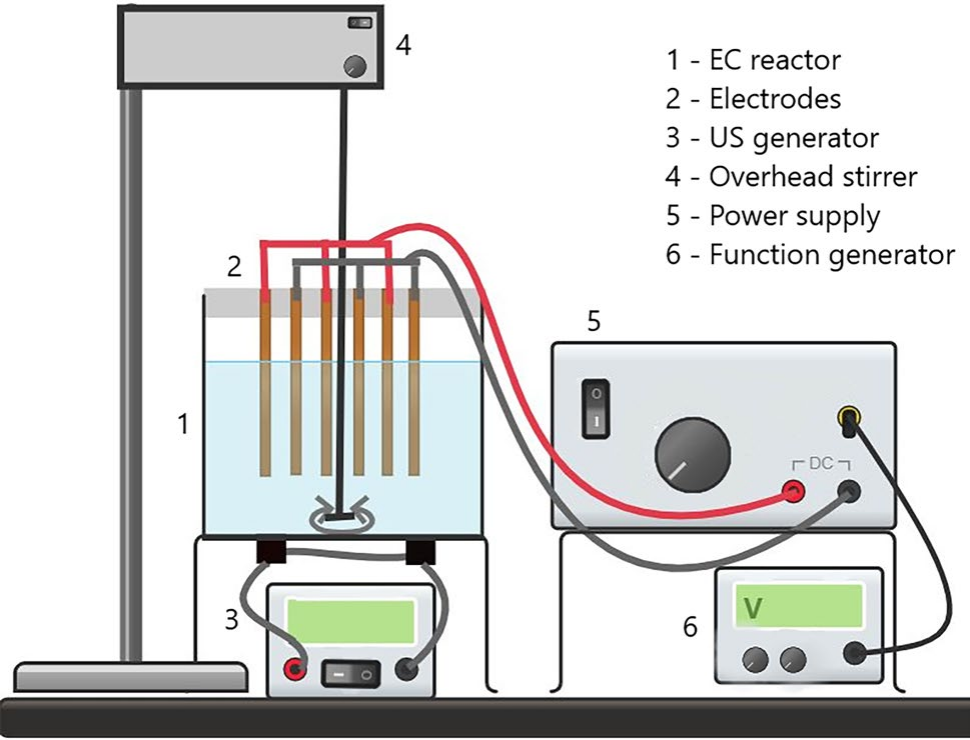
Schematic view of the experimental set up.

All the experiments were carried out in a 4-L plexiglass (polymethylmethacrylate, PMMA) reactor, as shown in Fig. 1. Four ultrasonic transducers were mounted on a metal plate at the bottom of the reactor and connected to the ultrasonic generator with a total nominal power of 480 W. The metal bottom plate was insulated with a thin, self-adhesive, transparent plastic film to prevent electrochemical reactions with the material. Six perforated metal plate electrodes (3 anodes and 3 cathodes) with a total active surface area of 98 cm^2^ were placed in the reactor 1 cm apart. The electrodes were connected to a 3000 W power supply (CSP-3000–120 from Mean Well, Taiwan) and controlled by the output voltage of the function generator (JT-JDS6600 from Joy It, Germany). Three different commercially available electrode materials were tested: iron (Fe; S355J2 + N alloy), aluminum (Al; EN AW-5754 alloy), and copper (Cu; Cu-ETP alloy). An overhead laboratory stirrer set to 150 rpm was used for mixing. A 3.5 L sample of model solution containing 10 mg/L imidacloprid (Boxer 200SL, Chromos Agro d.d.) and 1 g of NaCl as the electrolyte was used for each experiment. The initial conditions (model solution quality parameters) were measured by an HI98198 multiparameter (Hanna Instruments, USA) and are shown in Table 3. Samples were taken at specific time intervals using a syringe and filtered through a 0.45 µm PES filter. In addition to the EC investigation, a series of experiments with US and EC combined were also carried out. These tests were conducted for 5 min using the same electrode materials, a maximum current of 20 A and a 25 kHz US frequency. The concentration of imidacloprid was determined via high-performance liquid chromatography (HPLC with UV/VIS SPD-20A detector). The conditions for the analysis are listed in Table 4.

**Table 3:** The initial parameters of the model solution.

Temperature	20 ± 0.5 °C
pH	7.2 ± 0.2
El. Conductivity	547 ± 17 µS/cm^2^

**Table 4:** The conditions of the HPLC analysis.

Gradient Elution	5% A 95% B		
A	95% I	5% H_2_O	0.3% HCOOH
B	5% ACN	95% H_2_O	0.3% HCOOH
p	69–70 bar		
T	40 °C		
λ	260 nm		
V	20 µL		

To determine the dominant removal mechanism of acetamiprid, additional analysis of the sludge formed in the experiment with Al electrodes and 20 A of applied current in 20 min of treatment time was performed. After the experiment ended, 15 min of sludge settling time was allowed, after which the treated water above the sludge was decanted. The sludge was collected and left to dry on a filter paper for two hours at room temperature, as drying at high temperatures could potentially influence the degradation of any remaining acetamiprid. Afterwards, the sludge was weighed and thoroughly mixed with a 100 ml of ultrapure water. 3 filtered samples of this mixture were than analyzed by HPLC to see if there is any acetamiprid present in the sludge, as it is water soluble. Additional COD and chloride measurements were carried out at the beginning and the end of this experiment using ready-made cuvette tests and the Nanocolor 500 D photometer.

## Results and discussion

The efficiency of EC water treatment is, in general, influenced by many parameters, including reactor design and flow mode, electrode material and spacing, current density, electrolysis time, initial pH, presence of a supporting electrolyte, mixing speed, temperature and composition of the treated water ^21^.

All three electrode materials tested were found to be efficient at removing imidacloprid from water. Complete degradation (below the detection limit) of imidacloprid was observed with the combination of Al electrodes, 20 A of applied current (current density of 2040 A/m^2^) and 20 min of treatment. Under the same conditions, a 96.51% and 87.04% reduction in the imidacloprid concentration was achieved with the Fe and Cu electrodes, respectively. Although the highest efficiency was achieved with aluminum electrodes, iron and copper electrodes showed higher removal efficiencies when a lower current density and/or a shorter treatment time were applied.

The efficiency of electrochemical treatment depends on the coagulant dosage and floc production, which are influenced by the applied current density ^36^. According to Faraday's law, the amount of metal hydroxides produced by anode dissolution increases proportionally with increasing current density, suggesting that the removal efficiency is proportional to the current density ^21^. However, a current density that is too high can lead to overdosing of the coagulant, which can result in restabilization of the flocs present in the reactor due to charge reversal on the surface of the particles. This study also confirmed that higher currents lead to better contaminant removal rates.

The results also showed that treatment time is a very influential parameter for the electrochemical degradation of imidacloprid. The formation of metal ions and metal hydroxides, which are coagulating species, increases with increasing reaction time. The longer the treatment time was, the more coagulants were produced, and the more time remained for floc formation. However, prolonged treatment leads to increased energy consumption and higher costs.

The removal mechanism therefore consists of several successive and recurring processes during electrocoagulation. Various electrolytic reactions take place at and near the electrode surface, leading to the formation of coagulants in the aqueous phase, the adsorption of imidacloprid on coagulants and its removal by sedimentation or flotation. In addition, the total available input energy of the power supply is also used to increase the temperature of the solution by heating the electrodes and breaking the covalent bonds in the imidacloprid structure. The breaking of these bonds creates various fragments of the original molecule, which have one or two free electrons that combine with metal cations, hydrogen ions or other species. In this way, new compounds are formed, which in turn are adsorbed onto the formed coagulant and settle on the bottom. This was confirmed for glyphosate ^28^, and an identical removal mechanism can be assumed for imidacloprid but should be confirmed by a corresponding analysis of the precipitation.

Considering a simplified energy balance, in which the total available energy (E_tot_) of the EC reactor originates from the power supply, the bond dissociation energy (BDE) required to break the bonds between the atoms in an imidacloprid molecule, the energy needed for the release of metal cations from the anodes (E_w_), the energy transferred to the solution as heat (E_T_), and the energy required for the electrolysis of water (E_H2O_) are taken into account, it can be concluded that sufficient energy is available to break the bonds of imidacloprid, leading to complete degradation.

The total available energy, in the case of maximum removal efficiency (Al electrodes, I = 20 A, t = 20 min, V = 40 V), is calculated as follows:

7 $$\begin{array}{c}{E}_{tot}=I\times U\times t=20\; A\times 40\; V\times 0.333\; h =266.6\; Wh =959.90\; kJ\end{array}$$

**Figure 2:**
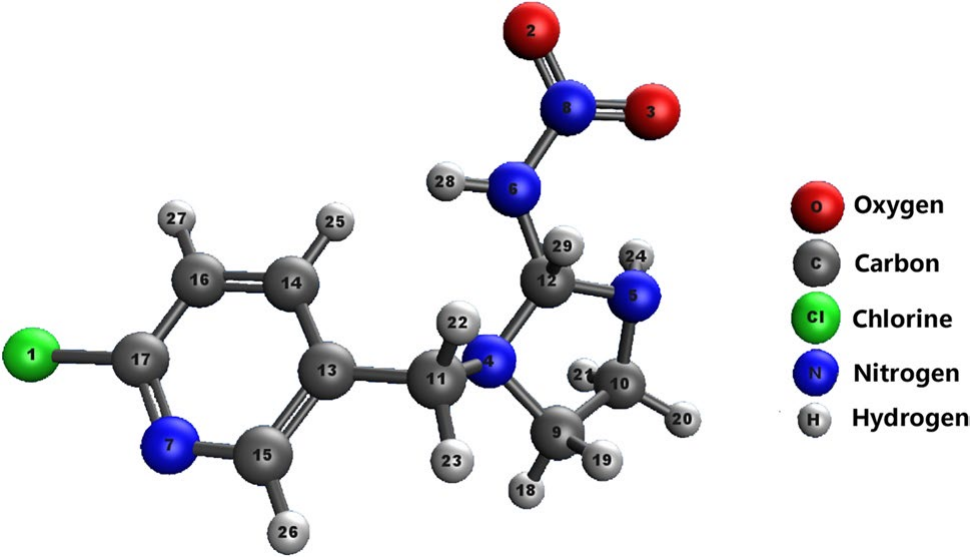
The structure of the imidacloprid molecule.

Figure 2 shows that the imidacloprid molecule (C_9_H_10_ClN_5_O_2_) has predominantly single carbon‒carbon (C–C), carbon–nitrogen (C–N), carbon-chlorine (C–Cl), and carbon‒hydrogen (C–H) bonds, while double bonds are present in the heterocyclic ring and with oxygen atoms. For this imidacloprid structure, the total enthalpy, *E*_*H*_, was calculated using the quantum chemistry program package Orca, release 5.0.4. (Turbomole GmbH, https://orcaforum.kofo.mpg.de/). A one-parameter hybrid version of the Perdew–Burke–Erzerhoff generalized gradient approximation with geometry optimization and analytical frequency calculation was used, for which the total molecular enthalpy of imidacloprid was calculated as follows:

$$ E_{H} = \, - {3}.{23} \times {1}0^{{6}}\; {\text{kJ}}/{\text{mol}} $$

Some of the homolytic binding enthalpies were calculated for the same imidacloprid structure: − 399.90 kJ/mol for the C11-C13 bond and − 797.585 kJ/mol for the C12-N6 bond (Fig. 2).

The minimum amount of energy required to transport an electron from a metal anode to a point in a vacuum just outside the solid surface is defined as the work function of a metal ^37^ and is available in the literature for different metals (e.g., the work function of aluminum is 4.08 eV, where 1 eV = 1.60217663 × 10^–19^ J). Zolotovitskii et al. ^38^ and Musumeci and Pollack ^39^ reported that the work functions of metals in a vacuum and in water are not the same, as the presence of water can lead to a significant decrease in the work function. Accordingly, the work function of aluminum in water is 3.00 eV ^38^.

Considering this value of the workfunction, the total energy required for the release of the *Al*^*3*+^ cations is:

8 $$\begin{array}{c}{E}_{W}=3\times 3.00\; eV\times \frac{m\left(Al\right)}{M\left(Al\right)}\times {N}_{A} =3\times 3\times 1.60217663\times {10}^{-19}\;J\times \frac{2.24g}{26.98 \frac{g}{mol}}\times 6.022\times {10}^{23}\frac{1}{mol}=72.08\; kJ \end{array}$$

where *m* is the mass of the released *Al* cations according to Faraday’s law, which in the case of maximum removal efficiency is:

9 $$\begin{array}{c}m= \frac{M\times I\times t}{e\times {N}_{a}\times z}=\frac{26.98 \frac{g}{mol}\times 20 \frac{C}{s}\times 1200 s}{1.6022 \times {10}^{-19}C\times 6.022\times {10}^{23}\frac{1}{mol}\times 3}=2.24\; g\end{array}$$

where *N*_*A*_ is the Avogadro constant, *e* is the charge of a single electron and *z* is the number of free electrons of the *Al* cation.

If the specific heat of water, *c*, is 4.184 kJ/kg °C, the energy used to heat the water in the reactor from 20 °C to 62 °C, as discussed subsequently, is:

10 $$\begin{array}{c}{E}_{T}=m\times c\times \Delta T=3.5\; kg\times 4.184\frac{{\text{kJ}}}{{\text{kg}}}\;^\circ C\times 42^\circ C =615.05\; kJ\end{array}$$

where *m* is the mass of the water in the reactor.

The standard enthalpy of formation of water is 285.83 kJ/mol. Since there is a 24 000 C charge (20 A × 1200 s), the amount of electrons participating in the hydrolysis is:

11 $$\begin{array}{c}n\left({e}^{-}\right)=\frac{Q}{e\times {N}_{a}}=\frac{24 000 C}{1.6022 \cdot {10}^{-19}C\times 6.022\times {10}^{23}\frac{1}{mol}}=0.2487\; mol\end{array}$$

Considering the half-reaction of water electrolysis, the number of electrons is twice as high as the number of hydrogen molecules produced and four times as high as the number of oxygen molecules produced. Thus, the number of moles of electrolyzed water is half the number of moles of electrons:

12 $$\begin{array}{c}n\left({{\text{H}}}_{2}{\text{O}}\right)=\frac{n\left({e}^{-}\right)}{2}=\frac{0.2487\mathrm{ mol}}{2}=0.1244\; mol\end{array}$$

which gives the total water enthalpy of the reactor:

13 $$\begin{array}{c}{E}_{{\text{H}}2{\text{O}}}=285.83 \frac{kJ}{mol}\times n\left({{\text{H}}}_{2}{\text{O}}\right)=285.83 \frac{kJ}{mol}\times 0.1244\; mol =35.56\; kJ\end{array}$$

Additionally, approximately 0.3 L of water, in total, melted from the ice containers that cooled the reactor. Considering the enthalpy of fusion of water to be 333.55 kJ/kg, the energy used to melt the ice (the heat from the surrounding air is neglected), E_H2O_^S^, is:

14 $$\begin{array}{c}{E}_{s}=333.55 \frac{kJ}{kg}\times m\left({{\text{H}}}_{2}{\text{O}}\right)=333.55 \frac{kJ}{kg}\times 0.30\; kg =100.07\; kJ\end{array}$$

Thus, this heat is transferred to the ice containers through the reactor walls. The energy available for breaking imidacloprid bonds, *E*_*H,A*_, is therefore:

15 $$\begin{array}{c}{E}_{H,A}={E}_{tot}-{E}_{W}-{E}_{T}-{E}_{H2O}-{E}_{s}=959.90-72.08-615.05 -35.56-100.07=137.15\; kJ\end{array}$$

For an imidacloprid concentration of 10 mg/L and a molar mass of 255.66 g/mol, the total amount of imidacloprid in the reactor was 3.911·10^–5^ mol. Considering the previously stated calculated molecular enthalpy, the total energy required for breaking all the imidacloprid bonds was:

16 $$\begin{array}{c}{E}_{H,tot}={E}_{H}\times n\left({C}_{9}{H}_{10}Cl{ N}_{5}{O}_{2}\right)=\left|-3.2326\times {10}^{6}\right|\frac{kJ}{mol}\times 3.911\times {10}^{-5}\;mol=126.43\; kJ\end{array}$$

Considering that 137.15 kJ of energy remains after the heating of water, melting of ice, release of cations and electrolysis of water and that 126.43 kJ are required for the complete degradation of the imidacloprid present, it can be concluded that the total energy available in the reactor is sufficient to achieve the maximum degradation and measured removal efficiency. An analogous energy balance can be considered for other electrode materials. However, differences in removal efficiency can be attributed to differences in the work functions of different anode materials and in the energy requirements of other processes that are not included in the energy balance. For example, if Cu is considered, its work function is 82.8 kJ, which ultimately does not leave enough energy to break all the bonds of the imidacloprid molecule. Assuming the formation of a new species, it is of course sufficient to break even a single bond in the molecule. Thus, energy balance calculations enable a better assessment of the actual energy required for the degradation of pollutants and are therefore useful tools for further process optimization by reducing energy consumption and associated costs.

To determine whether imidacloprid is actually degraded (mineralized), COD (chemical oxygen demand) measurements were carried out for the experiment that yielded the highest efficiency. The results showed a 56% reduction in COD, indicating that the chemical bonds in imidacloprid molecules, are indeed broken (imidacloprid is degraded to smaller molecules). In addition, the generated sludge was collected and analysed for the presence of imidacloprid. Only 0.6% of the total mass of imidacloprid removed was found in the sludge, demonstrating that degradation of imidacloprid is the predominant mechanism, as opposed to physical removal by flocs formed.

### DoE results

**Figure 3:**
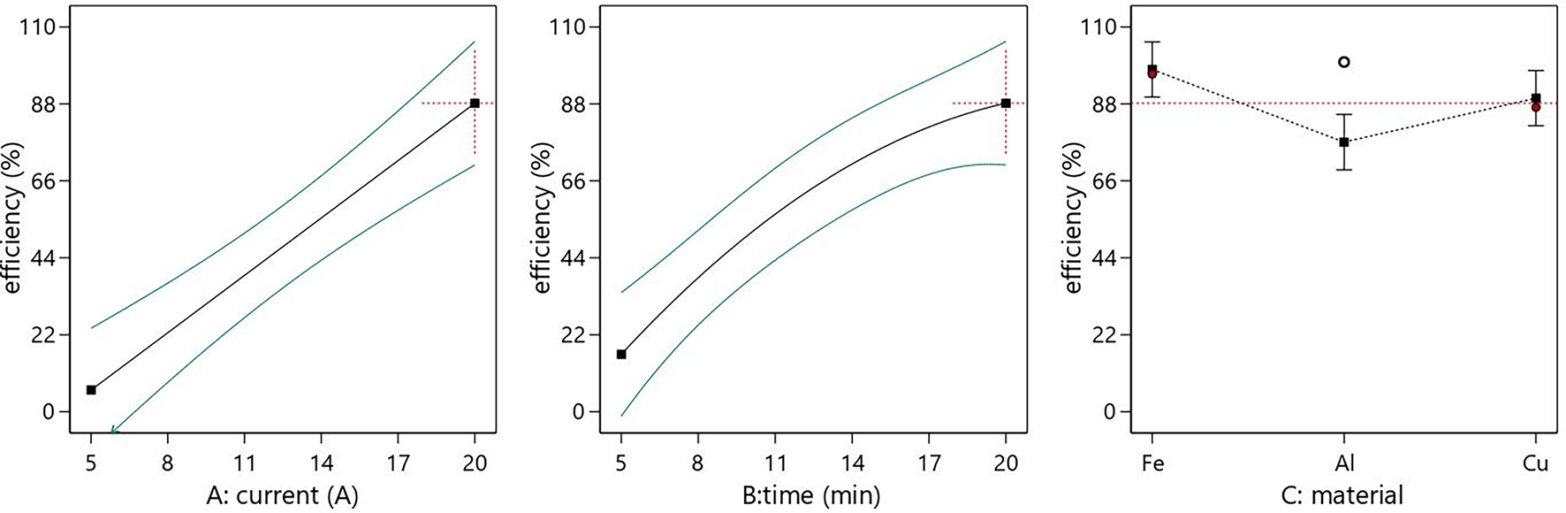
The effect of each factor on the efficiency of the process.

Analysis of variance (ANOVA) was used for the statistical evaluation of the obtained results. A reduced quadratic model was selected for further analysis. The significant factors and interactions were determined based on a significance level of 0.05. A p value of less than 0.05 indicates that the variable has a significant effect on the response, while a p value greater than 0.1 implies that the variable has little effect on the response ^40^. The ANOVA results, presented in Table 5, confirmed the significance of the model, as did all the included factors—material (C), applied current (A) and treatment time (B). The effect of each factor on the efficiency of the process is presented in Fig. 3. A and B had significantly lower p values than did the electrode material, and the interaction between these two factors was also significant. It can be concluded that the higher the current strength and/or the longer the treatment time are, the greater the efficiency, regardless of the electrode material used. These results are consistent with the findings of previous studies in which all electrode materials used in this work achieved high efficiency in removing pesticides. The wide range of response values suggests that a smaller range of factor values with higher values for each factor level should be used in future work to gain a better understanding of factor interactions.

**Table 5:** ANOVA results.

Source	Sum of	df	Mean	F value	p value	
Model	23,548.21	6	3924.70	13.4248	< 0.0001	Significant
A-el.current	10,430.01	1	10,430.01	35.6769	< 0.0001	
B-time	6480.15	1	6480.15	22.1660	< 0.0001	
C-material	2190.12	2	1095.06	3.7458	0.0391	
AB	3441.53	1	3441.53	11.7721	0.0023	
B^2^	1006.40	1	1006.40	3.4425	0.0764	
Residual	6723.96	23	292.35			
Lack of Fit	6666.79	20	333.34	17.4908	0.0185	Significant
Pure Error	57.17	3	19.06			
Cor Total	30,272.18	29

**Figure 4:**
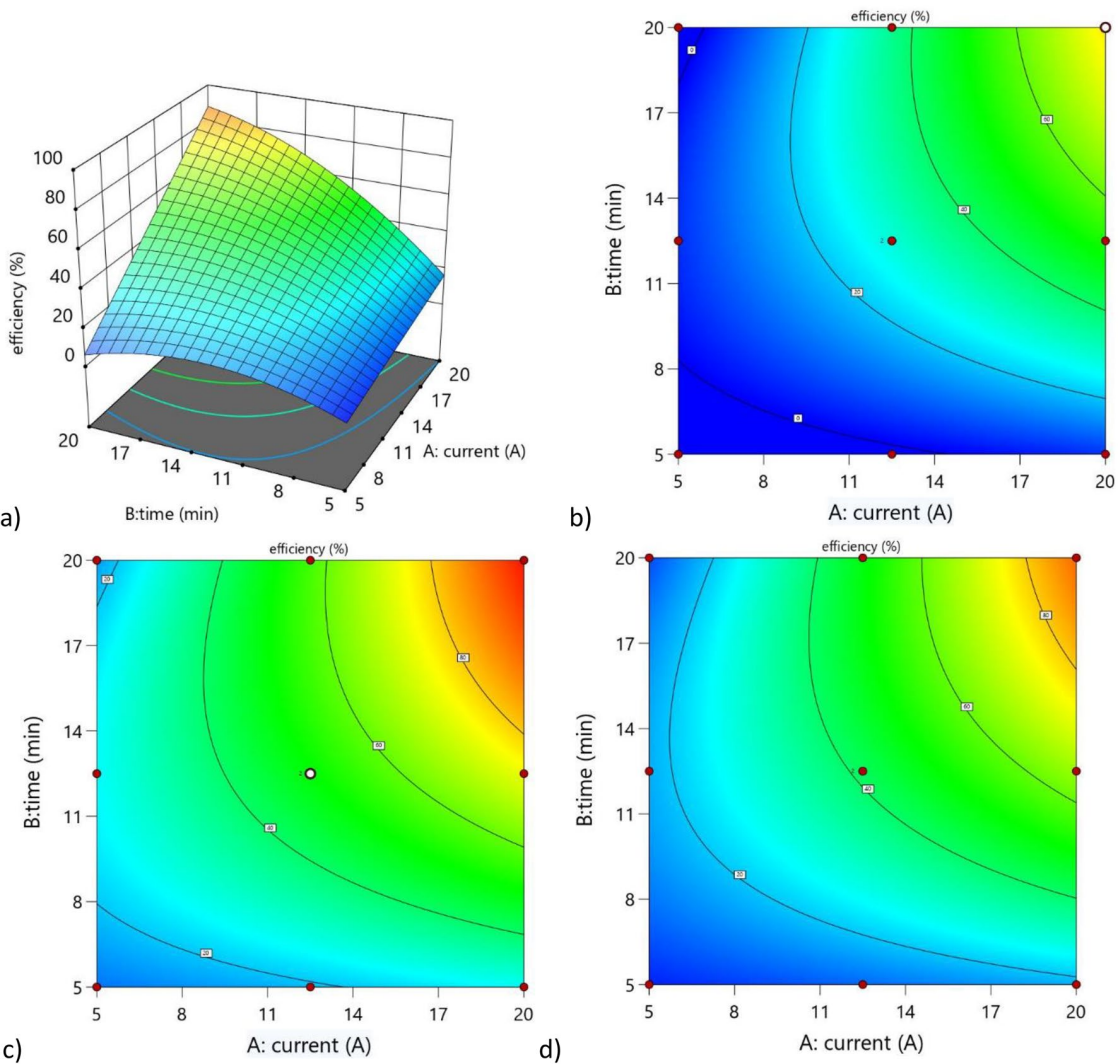
(**a**) Response surface of the obtained model (average over), (**b**) contour plot for the Al electrode, (**c**) contour plot for the Fe electrode, and (**d**) contour plot for the Cu electrode.

The three-dimensional response surface plot as a function of applied current and treatment time as well as contour plots for each electrode material are shown in Fig. 4. The model is described by the following equations in terms of the actual factors for each electrode material: Eq. (17) for Al, Eq. (18) for Fe and Eq. (19) for Cu.

17 $$Eff \left(\%\right)=-28.617-0.554\times A+4.021\times B+0.301\times A\times B-0.210\times {B}^{2}$$

18 $$Eff \left(\%\right)=-7.840-0.554\times A+4.021\times B+0.301\times A\times B-0.210\times {B}^{2}$$

19 $$Eff \left(\%\right)=-16.048-0.554\times A+4.021\times B+0.301\times A\times B-0.210\times {B}^{2}$$

The model was verified by repeating the experiments for the central design points (12.5 A and 12.5 min). This resulted in a difference of less than 5% for each of the electrode materials.

### The effect of temperature

The temperature during the EC can affect the electrode solubility, floc formation and conductivity, as well as the rates at which the reactions occur ^41^. To achieve a maximum current of 20 A, a higher voltage was needed to overcome the electrical resistance of the solution. This led to an increase in the temperature of the electrodes and thus to an increase in the temperature of the solution. Even though the reactor was cooled with ice containers on all sides, the temperature of the solution could not be kept constant. At the highest current, the temperature of the solution increased to 62 °C.

The effect of temperature on the degradation rate of imidacloprid was investigated, and it was found that the degradation efficiency increased with increasing temperature up to an optimum (30 °C), beyond which no further increase in degradation was observed ^42^. Patil ^43^ showed that imidacloprid degradation in wastewater by hydrodynamic cavitation was generally greater at elevated temperatures but decreased with increasing temperature (12.85%, 12.69%, and 12.54% at operating temperatures of 34 °C, 39 °C, and 42 °C, respectively). The complete degradation of imidacloprid by using hydrogen peroxide occurred after 120 min, compared to the 60 min needed for the Fenton process ^43^. Imidacloprid is quite stable in acidic and neutral water, but under alkaline conditions, its hydrolysis is enhanced by higher temperatures ^44^.

The specific mechanisms underlying the temperature dependence of imidacloprid decomposition are not fully understood. However, it is known that temperature can affect the kinetics of chemical reactions, and this is likely a contributing factor to imidacloprid decomposition. Higher temperatures could lead to different chemical reaction pathways, potentially resulting in different byproducts of the decomposition of imidacloprid when other substances are present in wastewater. However, further research is needed to fully elucidate the underlying mechanisms involved.

### The effect of ultrasound

A set of experiments in which continuous ultrasound (US) was applied simultaneously during EC treatment showed an increase in process efficiency compared to that of EC treatment alone under the same conditions (5 min, 20 A). When Al and Cu electrodes were used, an increase of 7% on average was observed, and with Fe electrodes, an increase in efficiency of 12% on average was achieved. The increase in efficiency can be attributed to the additional energy of the ultrasonic cavitation that is transferred to the water. The effective energy of the ultrasonic cavitation was measured with an analog cavitation intensity meter (Cav-meter-2) from the MRC Lab (Israel). The measurements of the effective energy showed that 45.7 W/L to 61.0 W/L (160 W to 214 W) of the nominally available 480 W was transferred directly to the water.

The higher efficiency is attributed to a synergistic effect caused by ultrasonic cavitation. The propagation of ultrasonic waves results in cycles of high and low pressure, causing the growth and collapse (implosion) of gaseous bubbles with very high local (hotspot) temperatures, high pressures, microjets and shockwaves ^45,46^. Cavitation also leads to the generation of highly reactive hydroxyl species ^47^. These hydroxyl radicals oxidize at the bubble–liquid interface, where the radical concentration is highest, while thermal and pressure-induced decomposition of imidacloprid occurs within and around the collapsing cavitation bubble. At a greater distance from the collapsing bubble, these degradation phenomena are not present ^45,48^. In general, the influence of ultrasound on the degradation rate should be considered in conjunction with the volume of the solution/reactor, the ultrasound frequency, the power and the sonication mode (continuous or pulsed). At higher frequencies, more bubbles with smaller diameters and reactive radicals are formed but with lower cavitation intensities. A lower frequency produces a smaller number of bubbles with larger diameters but with stronger implosions and thus greater pressure and temperature effects ^45,49^. All of these findings are specific to a certain threshold level above which efficiency decreases; thus, a higher or lower frequency does not necessarily indicate higher or lower efficiency. This approach also applies similarly to ultrasonic power since the formation of a large number of bubbles in the solution reduces the transfer of ultrasonic energy to water due to increased acoustic impedance ^49^. Additionally, the effect of mechanical cleaning of the electrodes occurs due to the applied US, which minimizes the formation of passive layers on the surface of the electrodes.

### Treatment costs

Operating costs, in terms of the electricity and materials used in the treatment process, for the maximum removal efficiency experiment are calculated as:

$$Operational\; costs=a\times {C}_{el}+b\times {C}_{EC}$$

where: *C*_*el*_ is the electricity consumption of the electrodes for 1 m^3^ of treated water (kWh/m^3^) and *C*_*EC*_ (kg/m^3^) is the electrode material used for 1 m^3^ of treated water. *a* is the average electricity price (0.15 EUR/kWh) according to the national tariff models and *b* is the average market price (with cutting and shaping) of used Al electrode alloy (5.38 EUR/kg).

The electricity consumption of the electrodes is calculated as:

$${C}_{el}=U\times I\times \frac{t}{V}=40 V \times 20 A \times \frac{0.333 h}{3.5 L}=76.11 \frac{kWh}{{m}^{3}}$$

where: *U* is the supplied voltage (V), *I* is the used current (A), *t* is the treatment time (h), and *V* is the volume of treated water (m^3^) during the treatment.

The consumption of Al electrodes (kg/m^3^), considering the Faraday's law, is

$${C}_{EC}=\frac{2.24 g}{3.5 L}=0.64 \frac{kg}{{m}^{3}}$$

resulting in total operating costs per volume

$$Operational\,costs=0.15 \frac{EUR}{kWh}\times 76.11 \frac{kWh}{{m}^{3}}+5.38 \frac{EUR}{kg}\times 0.64 \frac{kg}{{m}^{3}}=14.86\frac{EUR}{{m}^{3}}$$

Taking into account that the initial concentration of imidacloprid is 10 mg/L, the cost of removal can also be expressed as 1.49 EUR/g of removed imidacloprid.

### Drawbacks of this study

There are some drawbacks in this study that should be addressed, such as the choice of supporting electrolyte, the temperature rise during treatment, the relatively high energy consumption and the lack of identification of the by-products of imidacloprid degradation. The choice of NaCl as the supporting electrolyte was based on previous studies, in which NaCl was mostly used. Due to the possible formation of disinfection by-products in the presence of chloride, chloride measurements were performed at the beginning and at the end of the experiment that yielded the highest efficiency. The results showed no significant change in chloride concentration (the initial and the final concentration were around 2.5 mg/L), indicating that disinfection by-products containing chloride ions were not formed. However, in our further research the use of the electrolyte will be avoided or NaNO_3_ will be used. Also, in future research, COD measurements will be performed in each experiment, as they provide an important insight into the complete mineralization of organic pollutants, such as pesticides. In addition, better temperature control will be ensured by implementing more efficient cooling. Although this process is very effective, it is quite energy intensive. A careful energy balance and process optimization are key to lowering energy consumption and the associated costs.

## Conclusion

EC has the potential to be a cost-effective and environmentally friendly method for treating wastewater, but it is important to carefully consider its limitations and explore ways to optimize its performance. In this study, complete degradation/removal of imidacloprid was achieved in 20 min with Al electrodes at a current density of 2040 A/m^2^, while 96.5% decrease of imidacloprid concentration was achieved with Fe electrodes when 680 A/m^2^ was applied to 3.5 L of a 10 mg/L imidacloprid solution for 20 min without pH adjustment.

Overall, high removal efficiencies were achieved with significantly shorter operating times and a much larger reactor volume than previously published papers. A shorter operating time, which requires the use of high current densities, inevitably leads to heating of the solution so that adequate heat dissipation from the solution can be considered. Additionally, it was found that the contribution of ultrasound to the removal efficiency was 7% for the Al and Cu electrodes and 12% for the Fe electrode on average. The simplified energy balance confirmed that the energy generated in the reactor was sufficient to achieve maximum imidacloprid degradation. US energy may represent additional energy for the degradation of the imidacloprid molecule, which can be included in the energy balance and overall optimization (energy/efficiency/time/cost) when US is considered. A more detailed methodology on the energy balance as a tool for energy consumption evaluation and EC process optimization is planned.

## Data Availability

The datasets used and/or analyzed during the current study are available from the corresponding author upon reasonable request.
